# How Machiavellian leadership affects employees' happiness at work: the role of psychological contract breach and coworker exchange

**DOI:** 10.3389/fpsyg.2026.1763335

**Published:** 2026-02-20

**Authors:** Hao Wu, Shuai Lu, Anle Liu

**Affiliations:** 1 School of Economics and Management, Liupanshui Normal University, Liupanshui, China; 2 School of Tourism and Wellness, Changzhou Vocational Institute of Industry Technology, Changzhou, China

**Keywords:** coworker exchange (CWX), happiness at work (HAW), hospitality industry, Machiavellian leadership (ML), psychological contract breach (PCB)

## Abstract

In the hospitality industry, where service quality heavily depends on frontline employees' emotional engagement and wellbeing, leadership behaviors play a critical role in shaping employees' workplace experiences. Despite the growing attention to dark leadership styles, limited research has examined how Machiavellian leadership (ML) affects employees' happiness at work (HAW) and the underlying psychological mechanisms in hospitality settings. Drawing on social exchange theory, this study investigates the impact of ML on employees' HAW, the mediating role of psychological contract breach (PCB), and the moderating role of coworker exchange (CWX). Study 1 employed a scenario-based experiment, recruiting 188 employees from four five-star hotels in China to examine the causal relationship between ML and employees' HAW by manipulating ML conditions. Study 2 was conducted among frontline employees of 11 five-star hotels in Southwest China, utilizing a three-wave survey (*N* = 483) to collect data. Structural equation modeling (SEM) was used to analyze the mediating role of PCB and the moderating effect of CWX in this relationship. The results indicate that ML significantly reduces employees' HAW. Furthermore, PCB mediates the negative impact of ML on employees' HAW, while CWX plays a significant moderating role in the relationship between ML and HAW. These findings extend the dark leadership literature by elucidating how ML disrupts social exchange processes and undermines employees' HAW in service-oriented organizations. From a practical perspective, the study highlights the importance of mitigating the negative consequences of ML through effective psychological contract management and by fostering high-quality CWX relationships in hospitality organizations.

## 1 Introduction

Happiness has become a widely discussed topic globally. Increasingly, people consider happiness to be the core goal of life (Farooq et al., 2024), and its importance is broadly acknowledged, transcending cultural, occupational, and educational backgrounds (Rastogi, 2020). As the understanding of happiness deepens, researchers in psychology and management have begun to focus on the applicability of this concept in the work environment, extending it to the workplace (Mousa and Chaouali, 2022; Salas-Vallina and Alegre, 2021). The overall joy employees experience in their work, the characteristics of the work, and their perceptions of the organization are collectively referred to as happiness at work (HAW; Salas-Vallina and Alegre, 2018). Focusing on employees' HAW is essential, particularly in labor-intensive industries such as the hotel sector, as employee happiness not only enhances customer satisfaction but also fosters long-term relationships between hotels and their customers (Akgunduz et al., 2023). Furthermore, research has shown that happy employees are typically more efficient, motivated, and loyal to their employers (Kaur and Kaur, 2024). Given the significance of the concept, further research is needed to identify the factors influencing employees' HAW (Dahiya and Raghuvanshi, 2021; Salas-Vallina et al., 2020).

However, it is concerning that employees in the hospitality industry often report lower levels of happiness compared to those in other industries, with lower levels of motivation for success as well (He et al., 2019). Especially in developing-country contexts, hotel human resource management tends to face greater challenges than in developed countries, where labor regulations, management systems, and employee protection mechanisms are generally more established (Wu et al., 2025; Jayasuriya et al., 2025). In such contexts, due to an ample labor supply and intense industry competition, many hotel organizations experience pressure to control costs and maintain efficiency, often at the expense of improving working conditions and providing adequate organizational support (Zopiatis et al., 2014). Prior research in developing regions further suggests that institutional constraints and limited organizational support mechanisms may intensify the effects of managerial and organizational practices on employee-related outcomes (Jan et al., 2025). Such conditions are likely to undermine employees' HAW. This phenomenon highlights the urgency of investigating the factors influencing HAW of hotel industry employees in developing countries. Existing literature suggests that leadership plays a crucial role in shaping employees' emotions, perceptions of their work, and organizational identification (Salas-Vallina et al., 2020). As a growing area of interest, leadership has been shown to impact both employee and organizational wellbeing significantly (Farooq et al., 2024; Salas-Vallina et al., 2020). Although previous studies have focused on the influence of leadership on HAW, most have concentrated on positive leadership styles, such as transformational leadership (Salas-Vallina et al., 2017), altruistic leadership (Salas-Vallina and Alegre, 2018), and spiritual leadership (Srivastava et al., 2022). However, with the increasing emphasis on short-term outcomes, leadership has also begun to show unethical tendencies (Salas-Vallina et al., 2020). Consequently, researchers should shift their attention to the effects of dark leadership. Unfortunately, despite growing interest in leadership styles, research on how dark leadership influences employees' HAW remains limited (Syed et al., 2022).

Although different forms of dark leadership exist, Machiavellian leadership (ML) warrants particular attention in the hospitality industry because of its strategic, self-serving, and instrumental nature. Prior studies have shown that ML exerts unique effects on hotel employees' behavior compared to narcissism and psychopathy (Zhuang et al., 2022), and it undermines job involvement through workplace incivility (Khalid et al., 2024). Moreover, evidence suggests that Machiavellian tendencies may be particularly salient and effective in the Chinese cultural context, where strategic calculation and relational maneuvering are more likely to be tolerated (Wu et al., 2024). As a result, ML behaviors may be more prevalent and influential in Chinese organizations, underscoring the importance of examining their implications for employees' HAW. Nevertheless, empirical evidence on ML remains scarce, despite its strong association with negative outcomes such as absenteeism and turnover intentions (Karatepe et al., 2023).

Furthermore, scholars have called for further investigation into the underlying mechanisms through which leadership influences employees' HAW (Mehmood et al., 2023). From the perspective of SET (Blau, 1964), employees interpret leaders' behaviors as signals of the extent to which mutual obligations are fulfilled, and respond accordingly in terms of attitudes. The psychological contract serves as a crucial bridge between employees and the organization by shaping perceptions of fairness and mutual obligations, which are often signaled and reinforced through leaders' behaviors (Wu et al., 2024). Previous studies have shown that the negative behaviors of toxic leaders often lead to unmet psychological expectations of employees, leading to psychological contract breach (PCB) (Hattab et al., 2022; Kayani and Alasan, 2021). At the same time, in the hospitality industry, which relies heavily on teamwork and interdependent service delivery, the quality of coworker exchange (CWX) becomes a critical situational factor shaping employees' work experiences (Ye et al., 2021). Accordingly, this study responds to this call by incorporating PCB as a mediating mechanism and examining CWX as a boundary condition in the relationship between ML and employees' HAW.

The aim of this study is to examine how ML affects employees' HAW in the hospitality industry and to clarify the mediating role of PCB and the moderating role of CWX in this relationship.

To achieve this aim, this study addresses the following research questions: (RQ1) Does ML negatively affect employees' HAW? (RQ2) Does PCB mediate the relationship between ML and employees' HAW? (RQ3) Does CWX moderate the relationship between ML and employees' HAW?

By doing so, this study makes several significant contributions to the literature on leadership and employees' HAW. Firstly, although there are some studies on the influence of leadership on employees' happiness, empirical evidence in this area remains relatively scarce (Ruiz-Rodríguez et al., 2023; Salas-Vallina et al., 2020). In particular, the influence of ML on employees' HAW has not been sufficiently explored. Thus, this study seeks to fill this gap by investigating how ML affects employees' HAW. Secondly, psychological contracts, as important drivers of employee motivation and organizational cooperation, have been widely studied in organizational research (Lee et al., 2024; Tekleab et al., 2020). Although PCB has been extensively researched in organizational behavior (Botha and Steyn, 2023), empirical studies on whether PCB mediates the relationship between ML and employees' HAW remain scarce. By exploring the mediating role of PCB in this relationship, this study offers a new perspective for the limited but growing body of research on employees' HAW. Finally, given the frequent and close social interactions among employees in the hotel industry, coworker relationships are especially important in this sector (Dong and Hon, 2025; Ye et al., 2021; Kim and Qu, 2020). Sherony and Green (2002) introduced the concept of CWX, emphasizing its crucial role within organizations. However, existing research has given limited attention to CWX, particularly its moderating effect. Specifically, no study has yet examined the moderating role of CWX in the relationship between ML and employees' HAW. Therefore, this study further examines how CWX moderates the impact of ML on employees' HAW, aiming to fill this research gap.

## 2 Theoretical background and hypotheses development

### 2.1 Theoretical background

Social exchange theory (SET), first proposed by Blau (1964), posits that norms of reciprocity, mutual trust, and perceived balance between contributions and returns govern social and organizational relationships. In organizational settings, leadership behaviors play a critical role in shaping employees' perceptions of exchange quality, fairness, and trust. When leaders violate reciprocity norms, employees are likely to reassess the exchange relationship, leading to negative emotional and attitudinal responses (Breevaart et al., 2014). ML, characterized by manipulation, self-interest, and a disregard for ethical considerations (Stradovnik and Stare, 2018), represents a leadership style that challenges the fundamental assumptions of social exchange. Such behaviors can undermine trust and signal an imbalanced exchange relationship (Chiaburu et al., 2013; Arias-Pérez et al., 2023), making SET particularly relevant for understanding the potential negative consequences of Machiavellian leadership for employees' HAW.

From the SET (Blau, 1964) perspective, psychological contracts capture employees' beliefs regarding mutual obligations between themselves and their organizations (Wu et al., 2025). Perceptions of psychological contract breach reflect employees' evaluations of whether exchange expectations have been fulfilled and thus represent an important mechanism linking leadership behaviors to employee outcomes (Mehraein et al., 2023; Robinson and Morrison, 2000). In addition, when CWX quality is high, employees may turn to reciprocity and support from colleagues as compensatory resources to cushion negative impacts (Sherony and Green, 2002).

### 2.2 Machiavellian leadership (ML) and on employees happiness at work (HAW)

The pursuit of happiness appears to be one of humanity's most enduring and resilient goals across time and cultures. In the organizational context, HAW is widely recognized as an expression of positive employee attitudes (Fisher, 2010). HAW is commonly conceptualized as a multidimensional positive employee attitude encompassing work engagement, job satisfaction, and affective organizational commitment (Salas-Vallina and Alegre, 2021; Fisher, 2010). Due to its positive impact on both individual and organizational wellbeing, HAW has attracted significant attention from organizational researchers (Farooq et al., 2024; Lyubomirsky et al., 2005; Meyer et al., 2002). Prior research indicates that higher levels of HAW are associated with greater engagement in organizational citizenship behaviors (Singh and Banerji, 2022). Additionally, higher levels of HAW have also been linked to improved employee mental health (Sudibjo and Manihuruk, 2022). Taken together, HAW represents a critical attitudinal outcome for organizational functioning.

Happiness has also been conceptualized in a variety of ways beyond the workplace. Some define it as subjective wellbeing, which refers to a person's overall positive evaluation of life (Diener et al., 1999). This view usually includes both feelings, such as positive and negative emotions, and cognitive judgments, such as life satisfaction (Haller and Hadler, 2006). Others, following a philosophical tradition, see happiness more as a matter of virtue, for example, being fair, honest, or courageous, and suggest that such traits often bring more positive emotions (Oravecz et al., 2020). In organizational research, attention has shifted to the workplace, where leadership is seen as especially important because it affects employees' motivation, trust, and commitment (Salas-Vallina et al., 2020; Botha and Steyn, 2023; Barile et al., 2015). With numerous definitions, it is crucial to clarify the meaning of happiness in this study (Salas-Vallina and Alegre, 2021). Accordingly, this study focuses on HAW as the operational definition, because it provides a comprehensive and context-specific representation of employees' positive work experiences and is more directly related to organizational outcomes than broader notions of happiness.

Current research has identified leadership as a key antecedent of HAW, particularly through its influence on employees' perceptions of the work environment and organizational support (Farooq et al., 2024; Fisher, 2010). Existing studies mainly emphasize the positive influence of positive leadership styles on employee HAW (Salas-Vallina et al., 2020). However, despite the growing focus on the impact of leadership on HAW and exceptionally positive leadership styles, research on dark leadership styles remains in its early stages, particularly regarding ML. Although some studies suggest that Machiavellian leadership may generate short-term benefits in highly competitive or high-pressure environments where leader and employee interests temporarily align (Deluga, 2001), the majority of research highlights its destructive long-term consequences, particularly for relational quality and employees' attitudes. ML, as a form of dark leadership, is characterized by manipulation, strategic deception, and a strong focus on personal gain, with leaders pursuing power and success by prioritizing self-interest over mutual obligations (Dahling et al., 2009; Paulhus and Williams, 2002; Stradovnik and Stare, 2018). Machiavellian leaders typically use manipulation, power plays, and deceit to advance personal goals, which undermines the trust and quality of relationships between employees and their leaders or supervisors, leading to adverse organizational outcomes (Karatepe et al., 2023; Arias-Pérez et al., 2023). As highlighted in a case study by Stradovnik and Stare (2018), the cunning and unethical behaviors of Machiavellian leaders lead to negative emotions and attitudes among employees toward the organization.

Drawing on SET, workplace relationships are fundamentally governed by norms of reciprocity and mutual obligation. When employees perceive fair, supportive, and trustworthy treatment from their leaders, they are likely to develop positive work-related attitudes (Kaur and Kaur, 2024). In contrast, when leaders behave in manipulative or self-serving ways, employees tend to interpret such behavior as a violation of mutual obligations, leading to the erosion of trust and perceptions of fairness (Arias-Pérez et al., 2023; Soral et al., 2022). Within this theoretical framework, employees' HAW reflects their overall evaluative response to the quality of ongoing social exchange relationships with leaders and organizational representatives. ML, characterized by self-interest, manipulation, and strategic deception, fundamentally violates these exchange norms by prioritizing personal goals over reciprocal obligations and relational fairness (Dahling et al., 2009). Such behaviors signal to employees that the exchange relationship is imbalanced and instrumental, prompting them to reassess the value and quality of their relationships with leaders and, by extension, with the organization. As part of this reassessment process, employees are likely to withdraw positive work-related attitudes grounded in reciprocal expectations, including work engagement, job satisfaction, and affective organizational commitment (Chiaburu et al., 2013; Moorman et al., 2025; Teven et al., 2006), which collectively constitute HAW. Consistent with SET, the continued erosion of reciprocal exchange under ML ultimately undermines employees' HAW. Therefore, this study proposes the following hypothesis:

H1: ML negatively impacts employees' HAW.

### 2.3 Psychological contract breach (PCB) as mediator

The psychological contract refers to the implicit, unwritten agreements between employees and employers based on mutual trust and expectations (Rousseau, 1995). It includes employees' expectations, commitments, and responsibilities toward the organization and the organizational expectations and commitments toward the employees (Rousseau, 1995). PCB occurs when employees perceive that the organization has failed to fulfill its implicit promises (Robinson and Morrison, 2000). As a negative work experience, PCB plays a critical role in shaping employees' attitudes and behaviors, as it reflects perceived violations of exchange expectations (Li et al., 2018). Accordingly, PCB provides an important lens for understanding how work environments and leadership behaviors influence employees' HAW (Stankevičiute et al., 2021). However, previous research on the impact of leadership on HAW has not addressed the mediating effect of PCB.

Drawing on SET, interpersonal and organizational relationships are sustained through reciprocal exchanges grounded in trust and mutual obligation (Blau, 1964). Within this framework, employees form expectations based on prior interactions that their organization and its representatives, particularly leaders, will honor implicit promises and maintain fair and balanced exchanges (Eisenberger et al., 2010). ML, characterized by manipulation, deceit, and self-serving behavior, fundamentally disrupts these reciprocal exchange processes by prioritizing personal interests over mutual obligations (Stradovnik and Stare, 2018). Such leader behaviors signal an imbalanced, instrumental exchange relationship, undermining trust and reciprocity in leader–employee interactions (Mehraein et al., 2023). Empirical research further indicates that destructive and toxic leadership behaviors frequently result in unmet psychological expectations among employees (Hattab et al., 2022), thereby increasing the likelihood that employees perceive PCB. Consistent with this view, Kayani and Alasan (2021) demonstrate that toxic leadership styles are positively associated with employees' perceptions of PCB. Therefore, employees exposed to ML are more likely to interpret their exchange relationship with the leader as violating implicit organizational promises, giving rise to PCB.

In addition, from a social exchange perspective, when employees perceive PCB, they interpret the exchange relationship with the organization or its representatives as failing to fulfill reciprocal obligations (Robinson and Morrison, 2000). In particular, employees may interpret leaders' manipulative and exploitative behaviors as signals of insufficient organizational support (Ahmed and Nawaz, 2015), thereby reinforcing perceptions that implicit promises have not been honored. Such perceptions prompt employees to reassess the value and quality of the exchange relationship and adjust their attitudes accordingly. In response to perceived violations of reciprocal obligations, employees are likely to withdraw from positive work-related attitudes grounded in expectations of fairness and support (Li et al., 2018). Empirical evidence consistently indicates that PCB is associated with lower levels of work engagement (Rayton and Yalabik, 2014), reduced job satisfaction (Robinson and Rousseau, 1994), and weakened affective organizational commitment (Lapointe et al., 2013), which together constitute HAW. Through this process of exchange reassessment and attitude withdrawal, PCB serves as a key psychological mechanism linking ML to diminished HAW. Therefore, this study proposes the following hypothesis:

H2: PCB mediates the negative effect of ML on HAW.

### 2.4 Coworker exchange (CWX) as moderator

CWX refers to the quality of interpersonal interactions among employees under the same leadership supervision (Sherony and Green, 2002). CWX reflects a horizontal relationship among colleagues based on mutual trust, respect, and support (Pfrombeck et al., 2020; Chiaburu and Harrison, 2008). In the hospitality industry, where daily work requires extensive coordination and emotional labor, CWX plays a particularly vital role (Kim and Qu, 2020; Ye et al., 2021). Furthermore, the quality of CWX plays a crucial role in the work environment, as it significantly affects employee attitudes and behaviors (Kim and Qu, 2020). For example, when CWX quality is higher within a team, employees are more inclined to engage in helping behaviors (Lin et al., 2024). High-quality CWX enhances employees' customer-oriented perspective-taking, improving service quality (Dong and Hon, 2025). In collectivist cultures, the positive effects of CWX are particularly pronounced, as these cultures emphasize collective welfare and teamwork (Dong and Hon, 2025). Therefore, studying CWX in the Chinese context is of significant importance. However, current research has rarely focused on the moderating effects of CWX, especially whether CWX can mitigate the harmful effects of dark leadership.

According to SET (Blau, 1964), employees rely on social resources such as support, trust, and reciprocity to maintain positive work-related attitudes, particularly when their primary exchange relationships are strained. Within organizations, leaders are typically the most salient source of exchange resources because they represent the organization and exert substantial influence on employees' attitudes and behaviors (Kurtessis et al., 2017). At the same time, coworker relationships constitute an important supplementary form of social exchange, especially in teamwork-intensive contexts such as the hospitality industry, where daily work requires close coordination and emotional labor (Kim and Qu, 2020). CWX represents a form of horizontal social exchange that provides employees with trust-based and supportive relational resources (Sherony and Green, 2002). High-quality CWX facilitates understanding, cooperation, and assistance among coworkers, promotes the development of workplace friendships, helps reduce psychological stress, and enhances employees' psychological safety (Mao, 2006). When employees are exposed to ML, the quality of vertical exchange relationships with leaders may deteriorate, resulting in reduced access to socio-emotional resources. Under such conditions, high-quality CWX can serve as an alternative source of social support (Kim and Qu, 2020), compensating for the lack of supportive leadership and buffering the negative impact of ML on employees' HAW. Empirical research further indicates that high-quality CWX strengthens employees' organizational identification and positive work-related attitudes by increasing mutual support and assistance among coworkers (Zhang et al., 2022). Accordingly, when CWX quality is high, employees are better able to maintain positive work-related attitudes after exposure to ML, thereby weakening the negative effect of ML on HAW. In contrast, when CWX quality is low, employees lack alternative exchange resources and are more vulnerable to the detrimental effects of ML. Therefore, this study proposes the following hypothesis:

H3: CWX moderates the negative impact of ML on employees' HAW.

**Figure 1:**
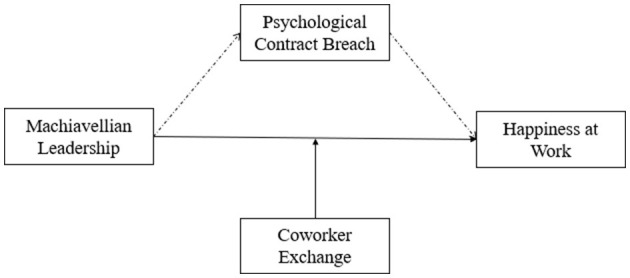
The research framework.

The research framework is shown in Figure 1.

## 3 Overview of research method

This study employs a mixed-methods approach to test the hypotheses. Specifically, Study 1 is a scenario-based experiment to examine the causal relationship between ML and employees' HAW. Scenario-based experimental designs are particularly useful for capturing respondents' reactions and intentions in specific situations, which have garnered considerable attention in the research literature (Elangovan et al., 2007). Study 2, on the other hand, is a multi-wave survey conducted with hotel employees in China, aimed at further validating the impact of ML on employees' HAW, with a particular focus on testing the mediating role of PCB and the moderating effect of CWX. To sum up, these two studies provide solid empirical support for our theoretical model and enhance the generalizability and robustness of the research findings.

### 3.1 Study 1

#### 3.1.1 Participants and procedure

This study involved four five-star hotels in China and recruited 188 frontline hotel staff with the consent of the human resource managers, using a convenience sampling approach commonly adopted in organizational behavior research. The total sample size (*N* = 188), comprising 98 participants in each group, exceeded the recommended threshold for detecting medium effect sizes in independent-sample *t*-tests (Cohen, 2013), ensuring sufficient statistical power. Before the experiment, participants were informed that their responses would be anonymous and that the data collected would only be used for academic research. Of the 188 participants, 64.4% were female, and 35.6% were male. Those aged between 26 and 35 accounted for the largest proportion, 67.6%. Additionally, 92% of the participants had been employed for over 2 years. The participants were randomly assigned to either the high ML scenario (*n* = 98) or the low ML scenario (*n* = 98). The experimental scenario was adapted from a study by Mudrack and Mason (1995).

##### 3.1.1.1 High ML condition

As a department employee in the hotel, you notice that the leader often conceals key information, particularly regarding team achievements, and takes credit for the success. When making decisions, the leader consistently prioritizes personal interests and manipulates team members to make decisions that benefit themselves. Over time, team members become aware of the leader's lack of transparency and the tendency to manipulate situations for personal gain, which leads to a decline in trust.

##### 3.1.1.2 Low ML condition

As a department employee in the hotel, you observe that the leader consistently maintains transparent communication with the team, sharing important information and publicly recognizing each member's contribution. When making decisions, the leader considers the team's overall interests, listens to everyone's opinions, and ensures that the decisions reflect the team's needs. As a result, team members feel respected and supported by the leader, which enhances trust.

Participants were given 10 min to read and imagine the scenarios to manipulate the experimental conditions. Following this, participants immediately completed a questionnaire that included the ML, PCB, and HAW scales and demographic information. Participants were informed that the questionnaire was anonymous and that their responses would be used solely for academic research. All scales used in this study were measured using a 5-point Likert scale (1 = Strongly Disagree, 5 = Strongly Agree). The ML scale was adapted from the 10-item scale developed by Mudrack and Mason (1995), with a Cronbach's α of 0.958. The PCB scale was based on the 5-item scale developed by Robinson and Morrison (2000), with a Cronbach's α of 0.911. The HAW scale was derived from the 9-item scale developed by Salas-Vallina and Alegre (2021), with a Cronbach's α of 0.944.

#### 3.1.2 Result

First, this study used a *t*-test to examine the effect of the manipulation of ML. The results indicated that the manipulation of ML had a significant impact, with participants in the High ML condition reporting significantly higher levels of ML compared to those in the Low ML condition [Mean_high_ = 3.948, SD = 0.507 vs. Mean_low_ = 1.829, SD = 0.380, *t*_(188)_ = 32.431, *p* < 0.001]. Second, the results showed that participants in the High ML condition evaluated HAW as significantly lower than those in the Low ML condition [*t*_(188)_ = −40.604, *p* < 0.001], indicating that ML reduced employees' HAW. Therefore, H1 was supported. Finally, the study also found that participants in the High ML condition reported significantly higher levels of PCB compared to those in the Low ML condition [*t*_(188)_ = 24.343, *p* < 0.001], suggesting that ML led to increased perceptions of PCB among employees.

### 3.2 Study 2

#### 3.2.1 Participants and procedure

Data were collected from frontline employees in 11 five-star hotels located in Southwest China, using a convenience sampling approach commonly adopted in organizational behavior research. From March to May 2025, 600 questionnaires were distributed, and 483 valid questionnaires were retrieved, with a response rate of 80.5%. The final valid sample size of 483 was well above the minimum recommended threshold of 200 for SEM analyses (Kline, 2023; Hair et al., 2017), ensuring sufficient power and robustness of the results. The demographic information of the participants is shown in Table 1. All participants were informed that their participation was voluntary, and their responses would remain anonymous and be used solely for academic research purposes. Data were collected at 3-time points to control for common method bias (Podsakoff et al., 2003). Demographic information and ML were collected at time-1. Employees' perceptions of PCB and CWX were assessed for time-2. Employees' HAW were measured at time-3.

**Table 1:** Demographic information.

**Category**	**Frequency**	**% (*n* = 483)**
**Gender**		
Male	220	45.5
Female	263	54.5
**Age**		
18–25	35	7.2
26–35	322	66.7
36–45	114	23.6
46 and above	12	2.5
**Tenure**		
2 years and less	36	7.5
3–6	218	45.1
7–11	183	37.9
11 years and above	46	9.5
**Education**		
High school or below	23	4.8
Junior college	65	13.5
Bachelor's degree	369	76.4
Graduate degree or above	26	5.4

The scales used for ML, PCB, and HAW were the same as those in Study 1. Specifically, Machiavellian leadership was measured using the 10-item scale adapted from Mudrack and Mason (1995) (Cronbach's α = 0.949), psychological contract breach was assessed using the 5-item scale developed by Robinson and Morrison (2000) (Cronbach's α = 0.922), and happiness at work was measured using the 9-item scale developed by Salas-Vallina and Alegre (2021) (Cronbach's α = 0.930). In addition, the CWX scale was based on the 6-item scale developed by Sherony and Green (2002), with a Cronbach's α of 0.905.

#### 3.2.2 Measurement model

Table 2 presents the factor loadings for each item of the variables. The results show that all factor loadings are greater than 0.5, indicating that no items need to be removed. In addition, the Average Variance Extracted (AVE) for each variable is greater than 0.5, and the Composite Reliability (CR) exceeds 0.9, suggesting good convergent validity and composite reliability. Table 3 displays the results of the Confirmatory Factor Analysis (CFA). The proposed model demonstrates good model fit indices (χ^2^/df = 1.760, RMSEA = 0.040, TLI = 0.970, CFI = 0.974, SRMR = 0.031). Furthermore, compared to other alternative models, the four-factor model proposed in this study (i.e., the proposed model) shows the best fit. Finally, the square root of the AVE for each variable is greater than the bivariate correlations between the variables, indicating good discriminant validity (Fornell and Larcker, 1981).

**Table 2:** Factor loadings, Cronbach's α, AVE, and CR for the variables.

**Variable**	**Item**	**Loading**	**Cronbach's α**	**AVE**	**CR**
ML	ML 1	0.766	0.949	0.584	0.947
	ML 2	0.777			
	ML 3	0.759			
	ML 4	0.788			
	ML 5	0.796			
	ML 6	0.831			
	ML 7	0.811			
	ML 8	0.809			
	ML 9	0.841			
	ML 10	0.834			
PCB	PCB 1	0.798	0.922	0.693	0.919
	PCB 2	0.807			
	PCB 3	0.865			
	PCB 4	0.845			
	PCB 5	0.846			
HAW	HAW 1	0.759	0.930	0.598	0.93
	HAW 2	0.761			
	HAW 3	0.672			
	HAW 4	0.78			
	HAW 5	0.813			
	HAW 6	0.73			
	HAW 7	0.831			
	HAW 8	0.779			
	HAW 9	0.824			
CWX	CWX 1	0.713	0.905	0.609	0.903
	CWX 2	0.788			
	CWX 3	0.808			
	CWX 4	0.703			
	CWX 5	0.841			
	CWX 6	0.818			

**Table 3:** Comparison of measurement models.

**Model**	**χ^2^/df**	**RMSEA**	**TLI**	**CFI**	**SRMR**
Proposed model^a^	1.760	0.040	0.970	0.974	0.031
Proposed model^b^	1.448	0.031	0.982	0.986	0.026
3-Factors model	4.521	0.085	0.861	0.877	0.090
2-Factors model	6.321	0.105	0.789	0.813	0.090
1-Factors model	8.087	0.121	0.719	0.750	0.105

3-Factors model: ML + PCB; 2-Factors model: ML + PCB + HAW.

^a^Without an unmeasured latent method construct.

^b^With an unmeasured latent method construct.

#### 3.2.3 Common method bias (CMB)

This study employed the Unmeasured Latent Method Factor (ULMF) approach to address the potential issue of CMB, as recommended by Podsakoff et al. (2003). This method involves adding an unmeasured latent factor to the measurement model to control for the variance in the observed variables that is attributable to the measurement method, thereby assessing whether CMB influences the relationships between the study variables. After incorporating the ULMF, the results of the CFA indicated that the inclusion of this latent factor did not significantly affect the model fit. Specifically, changes in fit indices such as RMSEA, CFI, and SRMR were less than 0.05 before and after the inclusion of the latent method factor. Therefore, based on the recommendations of Bagozzi and Yi (1990), CMB does not pose a threat to the validity of the findings in this study.

#### 3.2.4 Correlations

Table 4 presents the means, standard deviations, and bivariate correlation coefficients of the study variables. The results indicate that ML is positively correlated with PCB (*r* = 0.549, *p* < 0.001), negatively correlated with HAW (*r* = −0.660, *p* < 0.001), and negatively correlated with CWX (*r* = −0.436, *p* < 0.001). Additionally, PCB is negatively correlated with HAW (*r* = −0.749, *p* < 0.001) and negatively correlated with CWX (*r* = −0.506, *p* < 0.001). Meanwhile, HAW and CWX exhibit a significant positive correlation (*r* = 0.679, *p* < 0.001).

**Table 4:** Means, standard deviations, and bivariate correlations.

**Variables**	**Mean**	**SD**	**1**	**2**	**3**	**4**
ML	2.500	1.047	**0.764**			
PCB	2.390	0.962	0.549^***^	**0.832**		
HAW	3.878	0.829	−0.660^***^	−0.749^***^	**0.773**	
CWX	3.824	0.853	−0.436^***^	−0.506^***^	0.679^***^	**0.780**

^***^p < 0.001. Bold values on the diagonal represent the square root of the AVE.

#### 3.2.5 Hypotheses verification

This study tested the hypothesized relationships using AMOS 24.0 and the maximum likelihood estimation method. The results indicated that the model demonstrated a good fit to the data (χ^2^/df = 1.760, RMSEA = 0.041, TLI = 0.960, CFI = 0.964, SRMR = 0.062). As shown in Table 5, ML was significantly negatively associated with employees' HAW (β = −0.281, *p* < 0.001), providing support for Hypothesis H1.

**Table 5:** Path coefficients.

**Variable**	**HAW**	
	β	**SE**
ML	−0.281^***^	0.03
PCB	−0.407^***^	0.037
CWX	0.274^***^	0.037
ML × CWX	0.247^***^	0.056
*R* ^2^		0.748

^***^p < 0.001.

This study examined the mediating effect of PCB between ML and employees' HAW through mediation analysis. The test was conducted using the “user-defined estimands” function in AMOS 24.0 and employed 5,000 bootstrapped resamples to generate 95% bias-corrected confidence intervals (CIs) for assessing the significance of the mediation effect, following the procedure recommended by Preacher and Hayes (2008). The results in Table 6 indicated that the indirect effect was significant (β = −0.227, *p* < 0.001, 95% CI = [−0.306, −0.163]), suggesting that PCB mediates the relationship between ML and HAW. Therefore, Hypothesis H2 was supported.

**Table 6:** Mediation analysis.

**Path**	**β**	**SE**	**Bias-corrected 95% CI**	
			**Lower**	**Upper**
Direct effect	−0.281^***^	0.047	−0.373	−0.190
Indirect effect	−0.227^***^	0.037	−0.306	−0.163

^***^p < 0.001.

**Figure 2:**
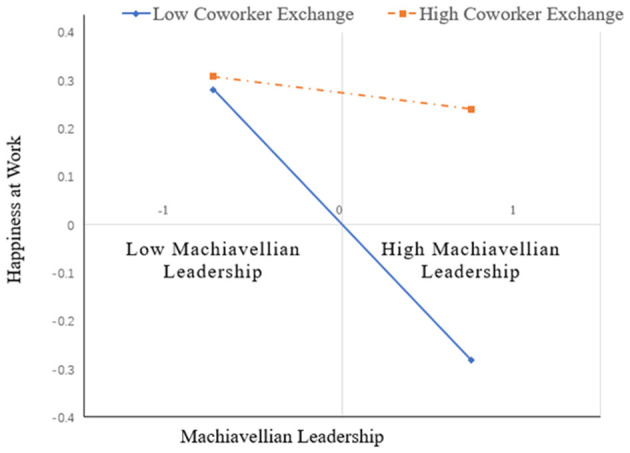
Simple slope analysis of the interaction between Machiavellian leadership and coworker exchange.

H3 posits that CWX moderates the negative impact of ML on employees' HAW. This study employed a mean-centering strategy to construct the interaction term between ML and CWX (Aiken and West, 1991), which reduces multicollinearity between the predictor and the interaction term, thereby improving the stability of the estimates. As shown in Table 6, the results indicate that the interaction term (ML × CWX) significantly affects HAW (β = 0.247, *p* < 0.001), thus supporting H3. A simple slope analysis was conducted to examine further the moderating effect of CWX on the relationship between ML and HAW. The results show that under high CWX conditions, the effect of ML on HAW was not significant (β = −0.034, 95% CI = [−0.168, 0.107], *p* = 0.619). However, under low CWX conditions, the effect of ML on HAW was significant and stronger (β = −0.528, 95% CI = [−0.673, −0.372], *p* < 0.001), indicating that when CWX is low, the negative effect of ML on HAW is more pronounced (see Figure 2).

## 4 Discussion and conclusions

Regarding H1, the findings confirmed that ML significantly decreases employees' HAW. This result aligns with previous research, which suggests that destructive leadership erodes trust and undermines positive employee attitudes (e.g. Karatepe et al., 2023; Stradovnik and Stare, 2018). Our results extend this understanding to the hospitality sector, showing that ML is particularly harmful in service-intensive contexts where employee wellbeing is central to organizational success. Although some scholars have found that ML has a positive impact in certain situations (Smith and Webster, 2017), in the long-term, such potential benefits are likely to be instrumental and short-lived, as they are achieved at the expense of trust and reciprocity (Liyanagamage and Fernando, 2023). For H2, the results demonstrate that psychological contract breach mediates the relationship between ML and HAW. Consistent with SET, this finding suggests that Machiavellian leaders' self-serving behaviors signal violations of exchange expectations, undermine trust and reciprocity (Mehraein et al., 2023), and, in turn, reduce employees' HAW. With respect to H3, the results indicate that CWX moderates the negative impact of ML on HAW. From the SET perspective, high-quality coworker exchange offers compensatory social resources, such as emotional support and reciprocity, that help employees cope with adverse leadership behaviors (Sherony and Green, 2002). This finding aligns with prior studies on the buffering role of coworker support (e.g., Zhao et al., 2023; Ye et al., 2021) and highlights the importance of considering horizontal social exchanges when examining employee HAW in hospitality settings. More broadly, these findings suggest that while CWX can buffer the negative effects of ML, it cannot fully offset the relational damage caused by sustained violations of reciprocity. It underscores the importance of addressing destructive leadership at its source rather than relying solely on horizontal support mechanisms.

### 4.1 Theoretical implications

This study makes several contributions to the existing literature. Firstly, as a form of dark leadership, ML often brings negative consequences by damaging organizational interests and hindering organizational development (Karatepe et al., 2023; De Hoogh et al., 2021). However, empirical research on the negative impact of ML remains scarce, especially within the hospitality industry (e.g., Karatepe et al., 2023; Huertas-Valdivia et al., 2022). By adopting a SET (Blau, 1964) perspective, this study moves beyond documenting negative outcomes. It provides a process-oriented, exchange-based account of how ML erodes positive work-related states. In doing so, the study advances existing research by shifting the focus from whether ML is harmful to why and how such leadership disrupts exchange relationships in service contexts.

Secondly, this study advances the literature by theorizing PCB as a key process mechanism through which ML undermines employees' HAW. PCB has long been an important area of research in organizational behavior, focusing on employees' perceptions of organizational commitment and expectations, and on how these expectations are fulfilled (Robinson and Morrison, 2000). However, limited research has examined how ML influences employees' HAW by affecting PCB. By integrating PCB into a Social Exchange Theory framework, this study clarifies how ML erodes employees' trust and perceptions of reciprocal obligation, thereby triggering a sense of PCB that leads employees to withdraw positive attitudes toward their work and organization. In doing so, the study shifts the emphasis from viewing PCB primarily as an outcome to conceptualizing it as a dynamic process mechanism that explains why and how ML ultimately diminishes employees' HAW, thereby enriching theoretical explanations of leadership-induced wellbeing erosion.

Third, this study found that CWX plays a significant moderating role in the relationship between ML and employees' HAW, responding to the suggestion by Breidenthal et al. (2020) that the quality of exchange (e.g., CWX) and its interaction with organizational contextual factors warrant further exploration. From an exchange perspective, when ML undermines vertical exchange relationships with leaders, high-quality CWX can compensate for depleted exchange resources by providing emotional support and instrumental assistance among coworkers. This finding broadens SET by illustrating that employees' HAW is not solely determined by leader-centered exchanges, but is also shaped by horizontal exchange processes that buffer the negative consequences of dark leadership. In this way, CWX is theorized as a critical contextual condition that constrains the extent to which ML erodes employees' HAW.

### 4.2 Managerial implications

The findings of this study provide several important implications for practice. Firstly, the findings of this study provide several important implications for practice. Firstly, given that ML often manifests through self-serving decision-making and manipulative interpersonal behaviors (Dahling et al., 2009), hotel organizations should remain vigilant toward leaders who exhibit such tendencies, as these behaviors are likely to erode employees' HAW. At the recruitment and promotion stages, hotels may incorporate personality assessments and situational judgment tests that emphasize empathy (Wu et al., 2024), reciprocity, and concern for employees' HAW, thereby reducing the likelihood of selecting leaders prone to exchange-disruptive behaviors. However, because selection mechanisms alone may not fully prevent ML, leadership development programs should move beyond generic training and instead integrate hospitality-specific case analyses that illustrate how manipulative leadership behaviors undermine positive employees' work attitude. In addition, establishing formal governance mechanisms, such as clear behavioral codes, confidential reporting channels, and accountability procedures, can help constrain opportunistic leadership behavior and reinforce norms of fairness and reciprocity.

Secondly, given that the negative effect of ML on employees' HAW operates through PCB, organizations should treat the management of employees' psychological contracts as a critical intervention point rather than a peripheral human resource practice. From a process perspective, PCB often emerges gradually as employees perceive misalignment between promised and actual work conditions, particularly under leaders who engage in self-serving or manipulative behaviors. To reduce the likelihood that such perceptions consolidate into a sense of breach, hotel organizations should actively monitor early signals of psychological contract strain, such as declining trust, increased cynicism, or disengagement among frontline employees. In practice, this requires not only clear communication and transparent decision-making but also timely clarification and recalibration of expectations related to workload, career development, and performance evaluation (Pfrombeck et al., 2020; Coyle-Shapiro et al., 2019). Importantly, psychological contracts in hospitality settings are often informal and dynamic (Wu et al., 2025). Accordingly, hotels should institutionalize regular opportunities for expectation review and renegotiation, especially following role changes, shift adjustments, or organizational restructuring. By intervening before perceptions of unmet obligations become entrenched, organizations can prevent the escalation of PCB and, in turn, protect employees' HAW even in the presence of problematic leadership.

Finally, the findings suggest that organizations can strengthen CWX as a compensatory resource when employees are exposed to destructive leadership behaviors that undermine vertical exchange relationships. When ML erodes trust and reciprocity between leaders and employees, supportive coworker relationships can serve as an alternative source of socio-emotional and instrumental resources, thereby buffering the negative impact on employees' HAW (Zhang et al., 2022). Rather than viewing coworker support as a general cultural ideal, hotel organizations should deliberately design work structures that facilitate high-quality CWX, particularly in contexts where leadership behavior is difficult to change in the short term. For example, when designed to promote sustained task interdependence and repeated interaction, team-based task allocation, peer mentoring systems for frontline staff, and cross-functional coordination mechanisms can increase opportunities for mutual assistance, trust-building, and shared problem-solving. By institutionalizing these horizontal exchange structures, organizations can reduce employees' dependence on a single exchange partner, enhance resilience to destructive leadership, and sustain employees' HAW even under adverse leadership conditions.

### 4.3 Limitations and recommendations for future research

Like any research, this study has its limitations. First, the data were collected from frontline employees of five-star hotels in Southwest China. While this sample represents the hospitality industry in China, the findings may not be directly generalizable to other cultural or organizational contexts. As Farooq et al. (2024) have suggested, cross-national analyses should be conducted, as cultural differences across countries may shape employees' perceptions of HAW. In addition, future studies could extend the investigation to other labor-intensive service industries, such as healthcare and retail. Secondly, while the two-study design combining a scenario-based experiment and a multi-wave field survey strengthens internal validity and robustness, both studies relied on convenience samples, which may constrain the generalizability of the results. Future research could employ probability sampling or more diverse organizational contexts to enhance external validity. Thirdly, this study primarily examined the mediating role of PCB and the moderating role of CWX. However, ML may influence employees' HAW through additional mechanisms. Future research could explore alternative mediators (e.g., leader–member exchange, emotional exhaustion) or moderators (e.g., perceived organizational support, psychological safety) to provide a more comprehensive understanding of the relationship between ML and HAW. In addition, future studies may also examine whether CWX moderates the relationship between ML and PCB, as coworker support might buffer employees' perceptions of contract breach under manipulative leadership. Finally, this study focused on ML as a dark leadership style, but future research could extend this investigation by incorporating other forms of dark leadership, such as narcissistic leadership.

## 5 Conclusions

The research examined the influence of ML on employees' HAW, with a particular focus on the mediating role of PCB and the moderating role of CWX. A mixed-methods approach was employed to test these relationships, combining a scenario-based experiment (Study 1) to establish causal inference and a three-wave field survey (Study 2) analyzed using structural equation modeling to validate the findings in the hospitality context. The results indicate that ML has a significant negative effect on employees' HAW and that PCB serves as a key mechanism underlying this relationship. Furthermore, the findings reveal that CWX moderates the impact of ML on HAW, such that high-quality CWX buffers the detrimental effects of ML on employees' HAW. Together, these findings clarify how and when ML undermines employees' HAW in service-intensive hospitality organizations.

## Data Availability

The raw data supporting the conclusions of this article will be made available by the authors, without undue reservation.
